# Supplementary material for: Distinctive whole-brain cell types predict tissue damage patterns in thirteen neurodegenerative conditions
Source: eLife. 2024 Mar 21;12:RP89368. doi: 10.7554/eLife.89368 (PMC10957173; doi:10.7554/eLife.89368)
Supplement: Supplementary file 2. [file elife-89368-supp2.docx]

**Supplementary File 2.** Origin of each disorder-associated t-statistic map.

| Disease | Study | Year of publication | Journal / Database | Diseased participants (n) | Controls (n) | Method | Pathology confirmed |
| --- | --- | --- | --- | --- | --- | --- | --- |
| LOAD | Harper et al | 2017 | Neurol Neurosurg Psychiatry | 68 | 73 | VBM | yes |
| EOAD | Harper et al | 2017 | Neurol Neurosurg Psychiatry | 29 | 73 | VBM | yes |
| PS-1 | Harper et al | 2017 | Neurol Neurosurg Psychiatry | 10 | 73 | VBM | yes |
| DLB | Harper et al | 2017 | Neurol Neurosurg Psychiatry | 25 | 73 | VBM | yes |
| FTLD-3RTau | Harper et al | 2017 | Neurol Neurosurg Psychiatry | 11 | 73 | VBM | yes |
| FTLD-4RTau | Harper et al | 2017 | Neurol Neurosurg Psychiatry | 17 | 73 | VBM | yes |
| FTLD-TDP43A | Harper et al | 2017 | Neurol Neurosurg Psychiatry | 12 | 73 | VBM | yes |
| FTLD-TDP43C | Harper et al | 2017 | Neurol Neurosurg Psychiatry | 14 | 73 | VBM | yes |
| PD | Zeighami et al | 2015 | eLife | 232 | 117 | DBM + ICA* | no |
| bvFTD | Dadar and Metz | 2023 | Zenodo | 70 | 133 | DBM | no |
| nfvPPA | Dadar and Metz | 2023 | Zenodo | 36 | 133 | DBM | no |
| svPPA | Dadar and Metz | 2023 | Zenodo | 30 | 133 | DBM | no |
| ALS | Dadar et al. | 2020 | Brain Communications | 66 | 42 | DBM | some |

*ICA* = tensor probabilistic independent component analysis*
