# Supplementary material for: Distinctive whole-brain cell types predict tissue damage patterns in thirteen neurodegenerative conditions
Source: eLife. 2024 Mar 21;12:RP89368. doi: 10.7554/eLife.89368 (PMC10957173; doi:10.7554/eLife.89368)
Supplement: Supplementary file 3. [file elife-89368-supp3.docx]

**Supplementary File 3.** Eighty cell-type related gene markers provided by the BRETIGEA R package.

| Gene marker name | Cell-type |
| --- | --- |
| AQP4, BMPR1B, EDNRB, FGFR3, GFAP, GJA1, SDC4, SLC1A2, GJB6, ALDH1L1, SLC25A18 | Astrocyte |
| C1QB, CD74, CXCR4, FOLR2, ITGAX, P2RX4, CCL3, CCL4, SLA, TLR1, TNFSF18, ARHGAP25, DHRS9, KBTBD8 | Microglia |
| ANXA3, CD34, CFH, IFI27, TM4SF1, SELE, TGM2, VWF, SDPR, IFITM1, ITIH5, APOLD1, TM4SF18, GPR116 | Endothelial cell |
| CHGB, CNR1, GABRA1, GABRB2, GAD2, KCNC2, OPRK1, RELN, SYT1, MYT1L, RIMBP2, ZMAT4, RAB3C, SYNPR, DLX6-AS1 | Neuron |
| FOLH1, MAG, MOBP, CLDN11, PLP1, KLK6, CNTN2, TF, UGT8, ST18, ERMN, XYLT1, SH3TC2, CNDP1, TMEM64 | Oligodendrocyte |
| GALR1, HAS2, NFYA, RGS13, TGFA, TNR, FPGT, MMRN1, CDH19, CA10, CRISPLD1 | Oligodendrocyte precursor cell |
